# Analysis of Genetic Diversity in Speckled Blue Grouper (*Epinephelus cyanopodus*) Based on Whole-Genome Resequencing Technology

**DOI:** 10.3390/ani16101551

**Published:** 2026-05-19

**Authors:** Xueqin Hu, Yukun Huang, Xiyin Zheng, Jinhui Wu, Chong Han, Hu Shu

**Affiliations:** 1 Key Laboratory of Conservation and Application in Biodiversity of South China, School of Life Sciences, Guangzhou University, Guangzhou 510006, China; huxueqqq@163.com (X.H.); 18718615843@163.com (Y.H.); zhengxyinnn@163.com (X.Z.); 2 Agro-Tech Extension Center of Guangdong Province, Guangzhou 510520, China

**Keywords:** *Epinephelus cyanopodus*, SNP, aquaculture genetics, population structure

## Abstract

To assess the current genetic status of cultured populations of the speckled blue grouper (*Epinephelus cyanopodus*) in China and to provide a scientific basis for the selective breeding of superior strains, this study utilized single nucleotide polymorphism (SNP) loci screened via whole-genome resequencing technology to analyze the genetic diversity and population genetic structure of 90 *E. cyanopodus* samples collected from six cultured populations in southeastern China. Comprehensive evaluation of various genetic diversity parameters indicated that all populations exhibited moderate genetic diversity with minor differences among them. Population structure analysis revealed weak genetic differentiation among populations, suggesting that anthropogenic introduction and translocation practices may be the primary factors contributing to the unclear population genetic structure. Additionally, this study identified a number of key genes associated with energy metabolism, muscle development, and environmental adaptation. The findings provide important theoretical foundations for the broodstock selection and scientific aquaculture of *E. cyanopodus*.

## Simple Summary

In this study, whole-genome resequencing technology was used to analyze the genetic diversity and population structure of six cultured populations of the speckled blue grouper (*Epinephelus cyanopodus*). The results showed that all populations exhibited moderate levels of genetic diversity, with weak genetic differentiation among populations. Some individuals displayed complex genetic backgrounds, which may be related to artificial introduction practices. The findings provide a scientific basis for the sustainable utilization of the germplasm resources of *E. cyanopodus*.

## 1. Introduction

Speckled blue grouper (*Epinephelus cyanopodus*), belonging to the family Epinephelidae under the order Perciformes, is a marine aquaculture fish of significant economic value. It is highly favored in the market for its firm flesh and delicious flavor [1]. This species possesses distinctive body color characteristics and is often introduced into aquariums for ornamental purposes. Due to historical overfishing, continuous habitat degradation, and slow population recovery, wild populations of *E. cyanopodus* have become scarce [2]. Following breakthroughs in artificial breeding technology for groupers [3], the marine aquaculture industry has developed rapidly, with the gradual promotion of industrial recirculating aquaculture models that enable artificial seedling rearing and large-scale production. Consequently, *E. cyanopodus* has become a “star species” in marine cage and pond aquaculture. However, along with the industry’s expansion, new concerns regarding germplasm resources have emerged. Anthropogenic introductions through translocation, or hybridization between escaped farmed individuals and other populations, may lead to genetic decline in offspring due to inbreeding, manifested as reduced population genetic diversity or slowed gene flow under natural conditions [4]. Genetic diversity serves as the foundation for species evolution and environmental adaptation; maintaining population genetic diversity is not only a safeguard for coping with current environmental changes but also crucial for future evolutionary potential [5]. Furthermore, understanding the genetic differences and levels of genetic diversity among populations can provide critical data to support genetic breeding efforts, helping breeders select and combine genetic traits to enhance production and economic benefits [6].

Currently, research on the population genetics of groupers has become relatively in-depth, with most studies based on mitochondrial DNA (mtDNA) markers and microsatellite DNA markers [7,8], revealing the population structure and evolutionary history of some grouper species. With advances in sequencing technology, whole-genome resequencing enables the acquisition of a large number of single nucleotide polymorphism (SNP) loci. Due to their advantages of high throughput, high coverage, high density, and operability, SNPs provide an important tool for population genetic research [9] and have been widely applied in studies on genetic diversity analysis and molecular marker-assisted breeding in aquatic animals [10]. A recent study by Wang et al. [11] focused on genome annotation and comparative genomics of this species, with no analysis of population genetic diversity. Existing studies have mostly focused on native populations in Southeast Asia, and a systematic genomic assessment of populations in Chinese waters, especially cultured populations, is still lacking. To date, no studies have been reported on evaluating the genetic diversity of *E. cyanopodus* populations and analyzing their germplasm resource status.

This study employs whole-genome resequencing technology and uses SNPs as molecular markers to analyze the genetic diversity and population genetic structure of six cultured *E. cyanopodus* populations from different geographic locations in China, elucidating the genetic background and current status of their germplasm resources. The findings will provide a theoretical basis for the selective breeding and scientific aquaculture of this species. Additionally, this study analyzes the characteristics of genetic differentiation to reveal the molecular mechanisms underlying population adaptation to different aquatic environments and identifies key genes and pathways with potential for applied utilization.

## 2. Materials and Methods

### 2.1. Sample Collection

From August 2024 to August 2025, tissue samples of *E. cyanopodus* broodstock used for breeding by local farmers were collected from six different geographic locations (Table 1), including the Zhangpu, Fujian population (ZP, *n* = 32); Dongshan, Fujian population (DS, *n* = 31); Shenzhen, Guangdong population (SZ, *n* = 33); Huidong, Guangdong population (HD, *n* = 30); Lingshui, Hainan population (LS, *n* = 30); Dongfang, Hainan population (DF, *n* = 32). Caudal fin clips were collected from all samples, preserved in ethanol, and stored in a cool, dark place. Fifteen individuals were randomly selected from each of the six populations for subsequent experimental analysis.

### 2.2. Extraction of Sample Genomic DNA and Construction of Resequencing Library

Genomic DNA was extracted using the Animal Tissue Genomic DNA Kit (Foregene Co., Ltd., Chengdu, China) following the manufacturer’s protocol. The extracted genomic DNA was dissolved in TE buffer. DNA purity was assessed using a Nanodrop spectrophotometer (Thermo Scientific, Waltham, MA, USA), and the DNA concentration was measured using the Qubit Flex fluorometric method (Thermo Scientific, Waltham, MA, USA). Approximately 50 ng of each sample was subjected to 1% agarose gel electrophoresis to check for DNA degradation. Subsequently, DNA library preparation and sequencing were performed by Wuhan OneMore Technology Co., Ltd. (Wuhan, China).

After the DNA samples passed quality control, library preparation was performed according to the methods and procedures of the Annoroad Universal EZ DNA Library Prep Kit for MGI V1.0 (CAT: AN210105-S), Annoroad Adapters for MGI, Set-T (N1-N12) (CAT: AN210108), and Annoroad DNA Cyclization Kit for MGI V1.0 (CAT: AN210107-S). The DNA was randomly fragmented into segments of 300–350 bp using an ultrasonic disruptor. Following fragment size selection, end repair and 3′ A-tailing were performed. Subsequently, sequencing adapters were ligated, and the target fragments were enriched via PCR amplification, followed by product quality control to complete library preparation. Sequencing was performed on the DNB-T7 platform at Wuhan OneMore Technology Co., Ltd. using the designated sequencing reagents. Paired-end (PE) sequencing was conducted, generating 150 bp paired-end raw reads. The sequencing data underwent filtering and deduplication including: evaluating the average quality score of reads using a sliding window and trimming low-quality bases (average quality below Q20) at both ends of each read; automatically detecting and removing adapter sequences from each read; filtering out low-quality reads (those with more than 40% of bases below Q15 or containing more than 5 Ns); and discarding reads with a final length of less than 50 bp after processing. Ultimately, high-quality clean reads were obtained for subsequent resequencing alignment and analysis.

### 2.3. Sequence Alignment and Variant Detection

Using the *E. cyanopodus* genome (Appendix A) assembled by our laboratory (unpublished) as the reference genome, the filtered sequencing data were aligned to the reference genome using bwa (v0.7.12-r1039) [12]. Alignment rates, coverage rates, and coverage depths were calculated to evaluate the alignment results. Based on the sequence alignment results, picard software (v1.124) [13] was used to remove PCR duplicates, and data with mapping quality below 20 were discarded. Finally, GATK software (v4.4.0.0) [14] was employed for population variant detection to obtain raw variant information, including SNPs and InDels. To obtain high-quality variant detection results, quality filtering was performed using GATK software (v4.4.0.0) with the following parameters: for SNPs, QD < 2.0 || FS > 60.0 || MQ < 40.0 || MQRankSum < −12.5 || ReadPosRankSum < −8.0; for InDels, QD < 2.0 || FS > 200.0 || ReadPosRankSum < −20.0. To ensure the robustness of the population analysis, the quality-filtered variant results were further filtered at the population level using VCFtools software (v0.1.16) [15], retaining only biallelic sites with a minor allele frequency (MAF) greater than 0.05, a missing rate less than 25%, and a minimum mean depth (min–meanDP) of 5. The parameters were set as follows: --maf 0.05; --min-alleles 2; --max-alleles 2; --min-meanDP 5; --max-missing 0.75. Annotation of the detected variants was performed using annovar software (v2016-02-01) [16] to obtain basic annotation information for variant sites, including gene and region information annotations.

### 2.4. Genetic Diversity and Population Structure Analysis

Based on the SNP information obtained above, population analysis was performed on 90 *E. cyanopodus* samples. To avoid the potential impact of linked loci on population structure analysis, plink software (v2.0) [17] was used to perform linkage disequilibrium pruning prior to population structure analysis, with the following parameters: --indep-pairwise 50 10 0.1. The pruning strategy employed a window size of 50 SNPs, a window step size of 10 SNPs, and a linkage disequilibrium coefficient r^2^ threshold of 0.1. PopLDdecay software (v3.43) [18] was used for genome-wide linkage disequilibrium (LD) decay analysis. VCFtools software (v0.1.16) [15] was used for genetic diversity analysis. Based on the filtered SNP loci, the following metrics were calculated: SNP density, nucleotide diversity (Pi), observed heterozygosity (Ho), expected heterozygosity (He), inbreeding coefficient (F), polymorphism information content (PIC), neutrality test (Tajima’s D), and population differentiation index (Fst). PIC ranges from 0 to 0.5, with higher values indicating greater informativeness [19]. Tajima’s D values greater than 0 suggest that populations may be experiencing strong balancing selection or have undergone bottleneck effects in small populations [20]. Phylogenetic analysis was performed using the neighbor-joining (NJ) method with VCF2Dis software (v1.47) [21]. Principal component analysis (PCA) was conducted using VCF2PCACluster software (v1.40) [22], based on the filtered SNP dataset, the first three principal components (PC1 and PC2) were calculated, the proportion of variance explained by each component was extracted, and a two-dimensional scatter plot was generated with PC1 and PC2 as the coordinate axes to visualize the genetic clustering patterns of individuals from the six populations. Admixture software (v1.3.0) [23] was used to simulate the ancestral origins of individuals with a preset K value ranging from 1 to 10, and the optimal K value was selected based on cross-validation error (CV error).

### 2.5. Selection Pressure Analysis

The two populations with the greatest genetic differentiation were selected as the research subjects. Using VCFtools software (v0.1.16) [15], population differentiation analysis and population polymorphism analysis were performed with a window size of 200 kb and a step size of 20 kb. Selective sweep analysis was conducted on these two populations by combining Fst and Pi. The intersection of the top 5% significant intervals from both methods was extracted as high-confidence candidate regions. Meanwhile, based on the direction of Log10(Pi Ratio) (in pop1_vs_pop2, a negative value indicates selection in pop1, while a positive value indicates selection in pop2), the selection signals were classified by population specificity, ultimately yielding the sets of genes under selection for each population and a comprehensive result. The selected regions were subjected to GO and KEGG enrichment analysis using clusterProfiler software (v3.6.1) [24].

## 3. Results

### 3.1. High-Throughput Sequencing Data Analysis

**Figure 1:**
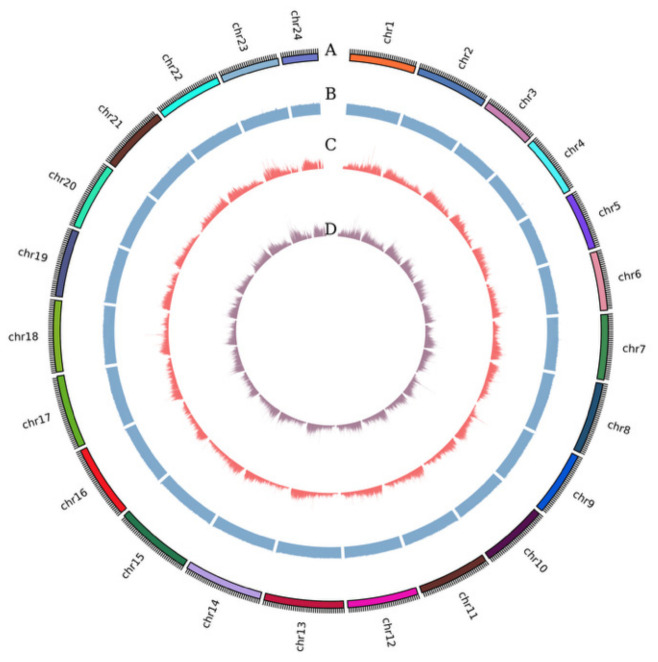
Genome-wide distribution of genetic variations. Ring A represents chromosomes; Ring B shows the distribution of GC content; Ring C indicates the number of SNPs per window; Ring D displays the number of InDel per window.

**Figure 2:**
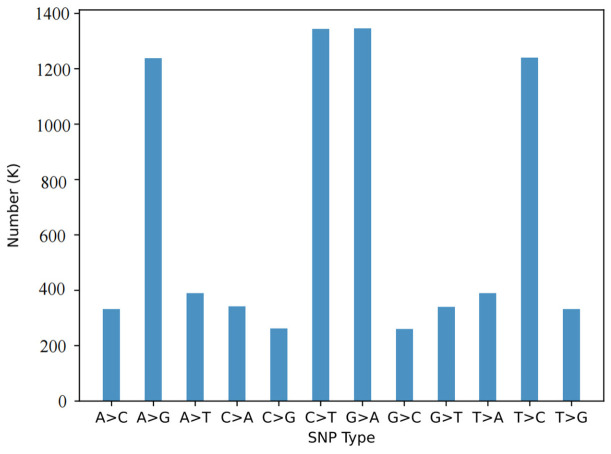
Classification of SNP types (transitions and transversions) identified in 90 samples.

Whole-genome resequencing was performed on 90 samples from six populations, generating a total of 1089 Gb of data, with an average data volume of 12.10 Gb per sample. The average alignment rate was 99.88%, the average coverage depth was 11.79×, and the average coverage rate was 98.91%. A total of 15,525,971 SNPs and 3,209,680 InDels were initially identified (Figure 1). After population-level filtering of the variant detection data, 7,812,666 high-quality SNP loci and 1,272,516 InDel loci were retained. To visualize the genome-wide distribution of variants, the Circos software (v0.69-9) was used to plot the distribution of SNPs across the 24 chromosomes. The results showed a relatively uniform distribution of SNP loci. The transition-to-transversion (Ts/Tv) ratio of all SNPs was 1.95 (Figure 2), which is slightly lower than the typical range of 2.0–2.1 reported for many teleost fish genomes but remains acceptable for population genetic analyses. Structural annotation statistics were performed on the detected high-quality SNPs (Table 2). The number of SNPs located in genic regions was 2,945,649, accounting for 33.70%. Among these, 3,980,269 SNPs were located in intronic regions, and 200,057 SNPs were located in exonic regions, including 125,469 synonymous mutations (62.72%) and 74,588 non-synonymous mutations (37.28%).

### 3.2. Genetic Diversity

Population genetic diversity is an important evaluation indicator of the current status of population germplasm resources. Multiple genetic diversity parameters were used to assess the genetic diversity of these six populations (Table 3). The study found that the SNP density was highest in the DF population at 7.6700/kb, and lowest in the DS population at 6.2094/kb. Overall, all populations exhibited similar levels of genetic diversity as indicated by comparable Pi values. Ho ranged slightly among populations, but notably, the DS population showed significantly lower Ho than He, suggesting a possible heterozygote deficit or inbreeding effect in this population. In contrast, most populations had Ho and He values close to each other, with F near zero. The PIC values of the six populations ranged from 0.2502 ± 0.1106 to 0.2859 ± 0.0902, indicating that the SNP markers used in this study possessed moderate informativeness. The Tajima’s D values were all greater than 0.

**Figure 3:**
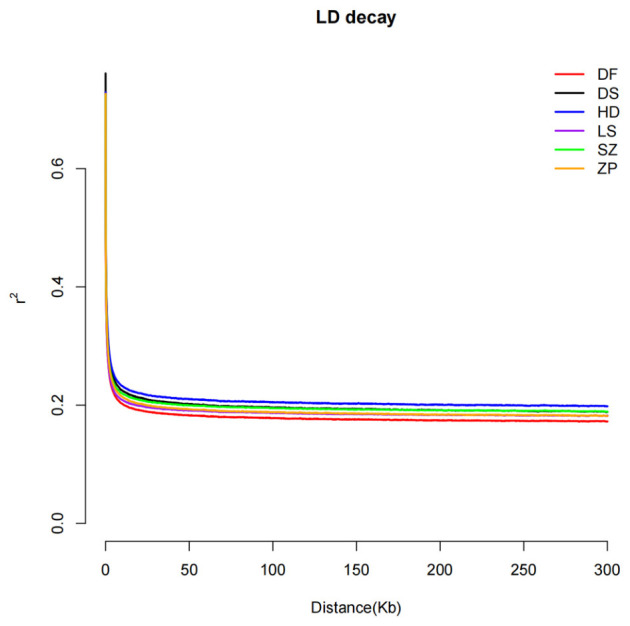
Linkage disequilibrium decay of different populations.

LD analysis was performed on the six populations, and the results are shown in Figure 3. Within the physical interval of 0–300 kb for the SNP loci, the LD decay rate was extremely rapid when the distance between markers was less than 20 kb, after which the decay rate gradually slowed. The differences in LD decay extent among the six populations were relatively small, with the half-decay distance ranging between 0.4 and 0.7 kb. The trend lines for the DS, LS, SZ, and ZP populations nearly overlapped.

### 3.3. Population Genetic Structure

**Figure 4:**
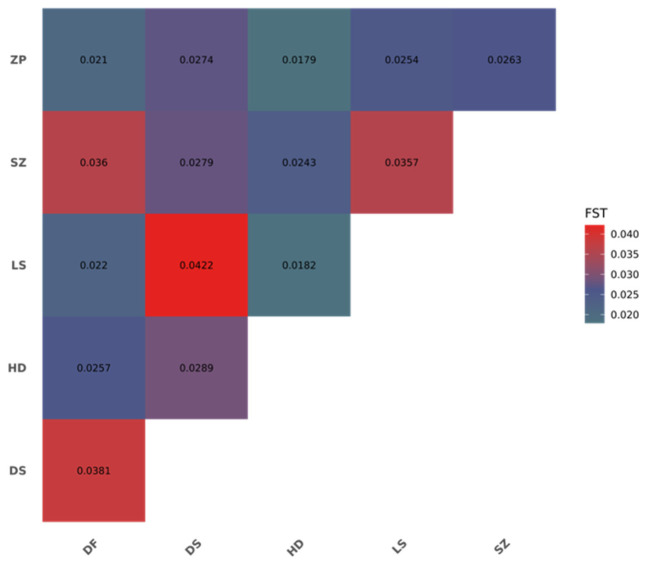
Genetic differentiation between the pairwise populations of *E. cyanopodus*.

To assess the degree of genetic differentiation among populations, the Fst was calculated for each pair of populations (Figure 4). The results showed that all pairwise Fst values were less than 0.05. The smallest Fst value was observed between HD and ZP (Fst = 0.0179), while the largest Fst value was observed between DS and LS (Fst = 0.0422). The Fst values between DS and the other five populations were slightly higher but still indicated very low differentiation. The Fst values between HD and the other five populations were very small. These results indicate that the degree of genetic differentiation among populations was relatively weak, with no significant genetic divergence. Furthermore, regardless of the geographic distance between populations, the Fst values did not show a corresponding increasing or decreasing trend, suggesting that there is no significant correlation between Fst and geographic distance.

**Figure 5:**
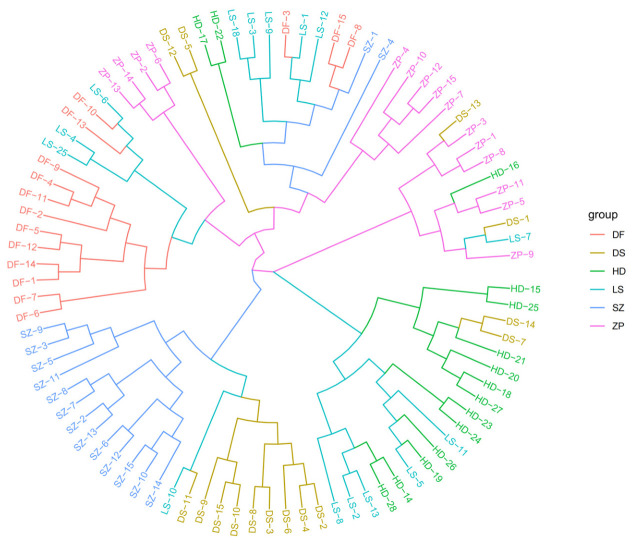
Phylogenetic tree of six populations.

**Figure 6:**
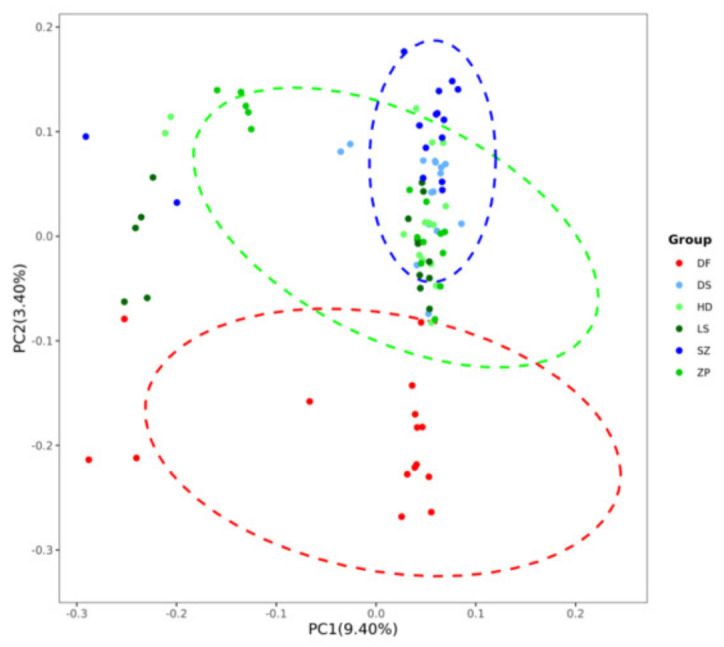
PCA scatter plot showing the distribution of the six populations. Each point represents a sample.

**Figure 7:**
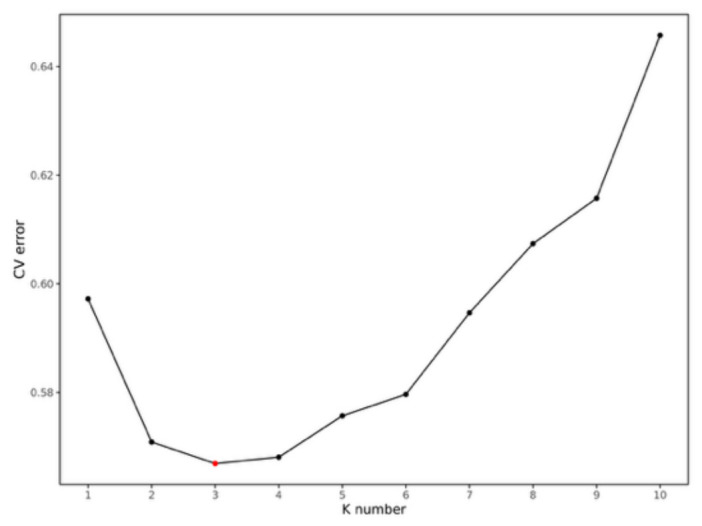
Line plot showing the CV error of the six populations.

**Figure 8:**
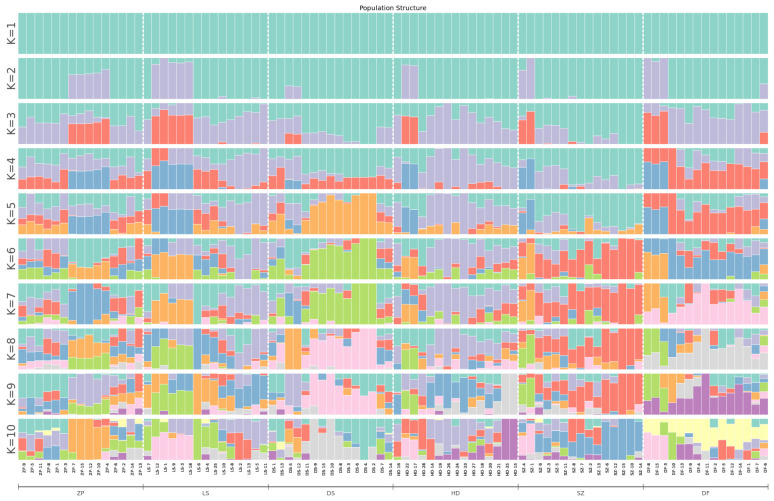
Bar plot of population genetic structure across different populations. The x-axis represents each individual and their assigned population. The y-axis indicates the estimated ancestry proportion derived from each simulation run. The color segments within each bar denote the assigned subpopulation, with their relative lengths corresponding to the proportional contribution of each ancestral source.

As can be seen from the topology of the phylogenetic tree (Figure 5), the clustering among samples exhibited a scattered pattern. Individuals from different populations were distributed across various small clusters in the tree, with no clear grouping based on geographic location. Most individuals from SZ and DS clustered together in one branch. Ten individuals from the DF population clustered into a small clade. Most individuals from HD were relatively concentrated in another branch, showing a close relationship with the ZP and LS populations. These results indicate that the population structure among some populations was not clearly defined and that they share close genetic relationships. PCA yielded similar results (Figure 6). The first principal component (PC1) explained 9.40% of the variance, and the second principal component (PC2) explained 3.40% of the variance. The six populations showed little difference along PC1 but exhibited some divergence along PC2. The populations were relatively close to each other in the PCA plot, with overlapping distributions. When K = 3, the CV error value was the smallest (Figure 7). The CV error value at K = 4 was close to that at K = 3. A lower CV error value indicates higher reliability [25], suggesting that K = 3 is the best-supported clustering solution, implying three major ancestral components rather than fully discrete biological groups. Based on the population structure bar plot (Figure 8), at K = 3, some individuals exhibited an admixture of two or more ancestral components. SZ and DS could be assigned to the same ancestral origin; most individuals in these two populations were predominantly green, sharing a high proportion of the same ancestral component, indicating relatively similar genetic backgrounds. The DF population was predominantly gray, representing a distinct ancestral origin. In contrast, the HD, ZP, and LS populations exhibited a mixture of multiple colors, indicating more complex genetic origins, suggesting that seedlings from these populations may have undergone more frequent cross-regional exchanges.

### 3.4. Identification of Selective Sweep Regions

**Figure 9:**
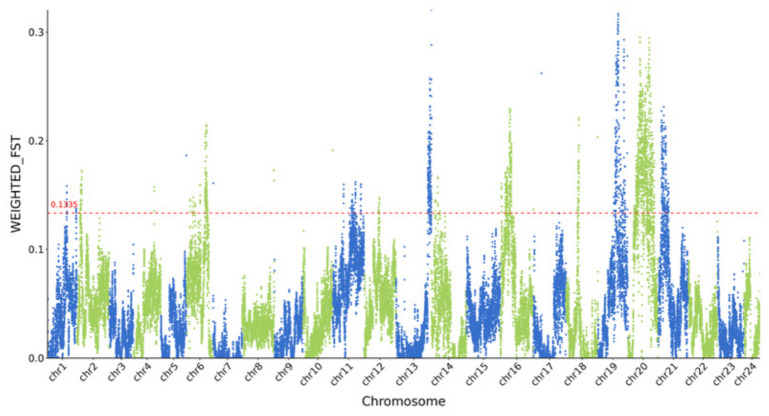
Manhattan plot showing the Fst values between the LS and DS populations. The red dashed line represents the top 5% threshold line.

**Figure 10:**
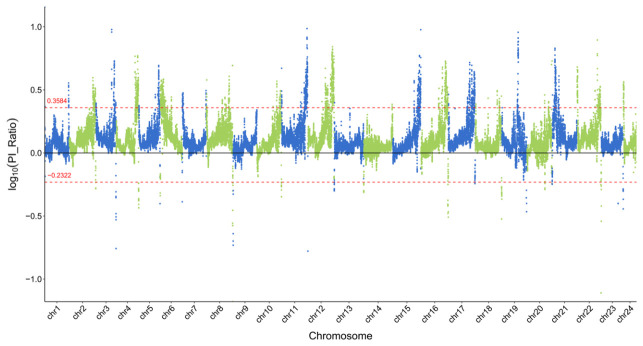
Manhattan plot showing the Pi ratio between the LS and DS populations. The positive and negative red dashed lines represent the top 5% threshold lines for population 1 and population 2.

**Figure 11:**
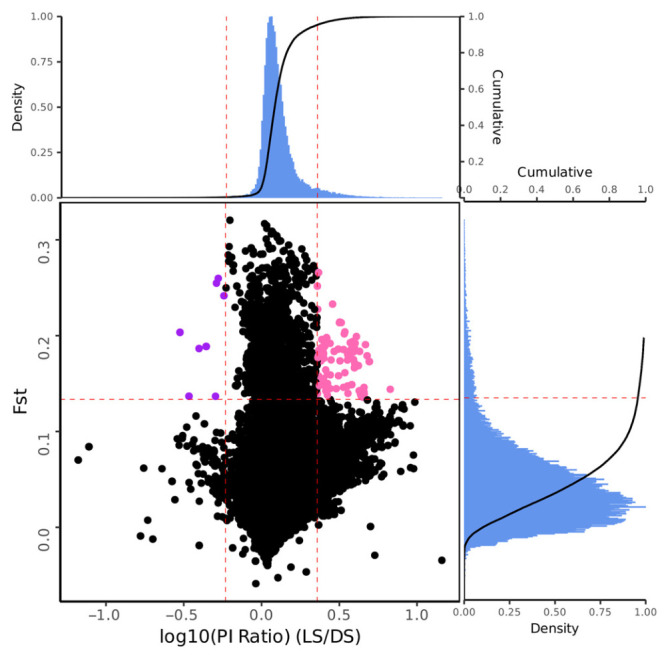
Joint analysis scatter plot based on Fst and Log10(Pi Ratio). The main plot is a volcano plot formed by Fst and Log10(Pi Ratio), where purple dots represent candidate regions of pop1 and pink dots represent candidate regions of pop2. The top subplot is a histogram and cumulative frequency curve of Log10(Pi Ratio), while the right subplot is a histogram and cumulative frequency curve of Fst.

**Figure 12:**
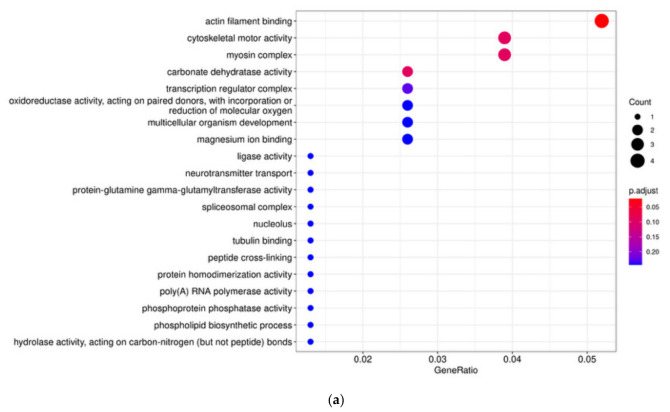

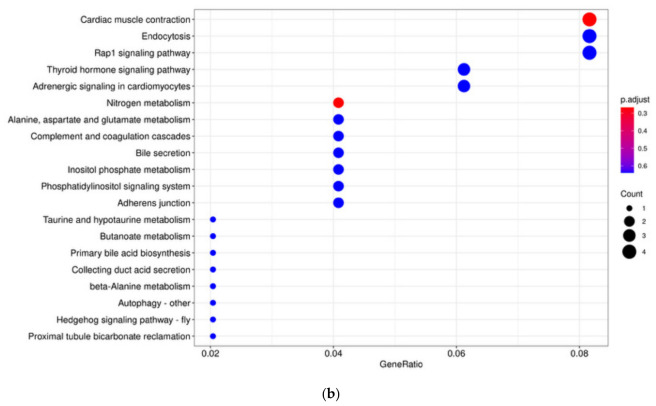
Enrichment analysis of candidate genes identified by the overlap of Fst and Log10(Pi Ratio) methods in the LS vs. DS population comparison. (**a**) GO enrichment analysis. (**b**) KEGG pathway enrichment analysis.

The LS and DS populations exhibited the largest pairwise Fst difference among all six populations (Fst = 0.0422). However, because this value is below the conventional threshold of 0.05 for significant population differentiation, our selective sweep analysis was conducted as a preliminary exploration rather than as a definitive test of selection. Using a combined Fst and Pi-ratio approach, we scanned for candidate genomic regions that might show signals of differential selection between these two populations. Given the low overall differentiation, the results should be interpreted as hypothesis-generating and require independent validation. The genome-wide distribution of Fst between the LS and DS populations is shown in Figure 9, and the distribution of Pi is shown in Figure 10. Regions where both Fst and Log10(Pi Ratio) values ranked within the top 5% were considered as selected regions for these two populations (Figure 11), and genes located within these regions were defined as candidate genes. Sixteen genes were identified in the LS population, mainly distributed on chromosomes 18, 19, and 20. Eighty-eight genes were identified in the DS population, mainly distributed on chromosomes 6, 8, 19, 20, and 21. Enrichment analysis was performed on all identified genes (Figure 12). GO enrichment analysis results indicated that these genes were mainly enriched in actin filament binding (GO:0051015), carbonate dehydratase activity (GO:0004089), cytoskeletal motor activity (GO:0003774), myosin complex (GO:0016459), and transcription regulator complex (GO:0005667). KEGG enrichment analysis results showed that these genes were mainly enriched in nitrogen metabolism (ko00910), cardiac muscle contraction (ko04260), and alanine, aspartate, and glutamate metabolism (ko00250).

## 4. Discussion

### 4.1. Genetic Diversity of E. cyanopodus Populations

Genetic diversity, as a key component of population genetics research, can assess the evolutionary potential of a population and is of great significance for the survival and reproduction of biological populations. Higher genetic diversity implies greater genetic differences among individuals, providing more potential adaptability for species in response to environmental changes [26]. Pi, as an important parameter for evaluating genetic diversity, has been widely used in genetic diversity studies of aquatic animals [19]. Heterozygosity, also known as gene diversity, reflects the degree of variation at genetic loci within a population. Lower heterozygosity indicates higher genetic consistency within a population; conversely, higher heterozygosity indicates higher genetic diversity [27]. Zhang et al. [28] used whole-genome resequencing technology to analyze the genetic diversity of the malabar grouper (*Epinephelus malabaricus*) in the South China Sea. Based on Pi (0.0023–0.0025), Ho (0.3328–0.3759), and He (0.3497–0.3520), their data indicated that the genetic diversity level of seven artificially cultured populations of *E. malabaricus* remained moderate, with low genetic differentiation among populations. Moreover, SNPs provided higher genomic coverage and could more accurately reflect the true genetic patterns. In the study by Luo et al. [29], the Ho values for the wild population of the orange-spotted grouper (*Epinephelus coioides*) in the South China Sea and the artificially bred population in Hainan were 0.7061 and 0.6875, respectively, and the He values were 0.4803 and 0.4215, respectively, indicating that the wild population still maintained relatively good genetic diversity. Dong et al. [30] used microsatellite molecular markers to analyze the genetic diversity of nine grouper species in the South China Sea. Ho ranged from 0.4615 to 0.6239, and He ranged from 0.3510 to 0.4754, indicating that the genetic diversity of all nine grouper species was at a moderate level. In the present study, the Pi values of the six *E. cyanopodus* populations ranged from 0.0022 ± 0.0014 to 0.0027 ± 0.0015. Ho (0.3241 ± 0.0708–0.3684 ± 0.0864) and He (0.3354 ± 0.0000–0.3716 ± 0.0001) were generally comparable, with Ho slightly higher than He in most populations, which was similar to other artificially cultured grouper species. Combined with the fact that F was mostly negative or close to zero (−0.0447 ± 0.2448–0.0810 ± 0.0687), these results indicate that the six *E. cyanopodus* populations possess moderate genetic diversity, with little difference among populations and similar genetic backgrounds. The level of inbreeding within populations was low, and random mating was relatively sufficient, with no obvious risk of inbreeding depression observed.

All six populations exhibited significantly positive Tajima’s D values (0.8559–1.6780), indicating a genome-wide deficit of rare variants and an excess of intermediate-frequency alleles. In population genetics, such a pattern can arise from multiple non-exclusive processes, including population bottlenecks, balancing selection, and admixture (population mixing) [31]. The Tajima’s D value of the SZ population (0.8559 ± 1.0302) was relatively low, which may indicate that it experienced a slightly different bottleneck intensity or timing compared to the other populations [32]. The criteria for PIC are as follows: PIC < 0.25 indicates low polymorphism, 0.25 < PIC < 0.50 indicates moderate polymorphism, and PIC > 0.50 indicates high polymorphism [33]. In this study, the PIC values of all populations (0.2502 ± 0.1106 to 0.2859 ± 0.0902) fell within the moderate polymorphism range, indicating that the selected SNPs possessed moderate genetic diversity and were suitable as genetic markers for population genetic analysis. LD decay reflects the degree of domestication and selection intensity in populations [34]. In this study, all six *E. cyanopodus* populations exhibited a rapid LD decay trend, with small differences in r^2^ values among populations and minimal genetic differentiation between populations. This indicates that the cultured *E. cyanopodus* populations had a low degree of domestication and similar genetic diversity. Among them, the DF population showed a slightly faster decay rate and had the highest SNP density (7.6700/kb), which could be due to a higher recombination rate, a relatively large effective population size, or a combination of these factors [35]. This pattern is consistent with the findings of studies on cultured orange-spotted grouper populations [28]. The DS population exhibited the lowest SNP density (6.21/kb) and Pi (0.0022 ± 0.0014), with Ho (0.3415 ± 0.0255) significantly lower than He (0.3716 ± 0.0001), indicating an excess of homozygotes. Additionally, it had the highest inbreeding coefficient F (0.0810 ± 0.0687), suggesting that inbreeding was most evident in this population. Its Tajima’s D value (1.6780 ± 0.8563) was the highest among the six populations, indicating that the DS population likely experienced a strong bottleneck effect. The bottleneck event led to the loss of rare variants, reduced the SNP density and Pi, and increased the proportion of medium-frequency alleles, resulting in a significantly positive Tajima’s D value. However, its LD decay was also relatively rapid, which may suggest that the effective population size of this population is recovering quickly after the bottleneck, or that a small amount of foreign gene flow has recently been introduced, thereby breaking long-standing linkage blocks. However, due to the rarity of wild *E. Cyanopodus* populations and the general lack of published genetic data for this species in natural habitats, we were unable to quantify how much genetic diversity may have been altered or lost from the ancestral wild gene pool as a result of aquaculture practices (founder effects, artificial selection, or genetic drift). The LS population had the highest Pi (0.0027 ± 0.0015) and a relatively high SNP density (7.6590/kb) with the most negative F value (−0.0447 ± 0.2448), indicating that this population had the richest genetic diversity and a pronounced excess of heterozygotes. Combined with the population structure analysis, the genetic background of individuals in the LS population was relatively complex with no obvious clustering. This may be related to management strategies that prioritize selecting distantly related individuals for broodstock breeding and avoid backcrossing to reduce inbreeding, leading to outcrossing or gene inflow that effectively maintained population genetic diversity [36,37]. However, frequent gene flow may cause interpenetration of the gene pools among populations, leading to homogenization of genetic composition and consequently exhibiting similar levels of diversity [38]. A limitation of this study is the lack of detailed breeding records. An ideal interpretation of the genomic results would require information on founder population sizes, number of selective generations, and selection intensity at each farm. Unfortunately, such records were not available for the six cultured populations examined here. This reflects a common challenge in aquaculture genetics research, particularly in small to medium-scale farming operations in China, where empirical broodstock management is often practiced without systematic documentation or molecular assistance. Consequently, we cannot provide precise numerical estimates of these parameters. Instead, we have interpreted the genomic data in light of the general management practices reported by the farmers: occasional exchange of fish among different bases, avoidance of backcrossing to reduce inbreeding, and phenotypic selection for larger body size. These practices are qualitatively consistent with the observed genetic patterns. Future studies that incorporate long-term breeding records or employ pedigree-reconstruction methods from high-density SNP data could overcome this limitation and enable rigorous demographic modeling.

### 4.2. Population Genetic Structure and Differentiation of E. cyanopodus

The CV analysis results indicated that K = 3 was the best-supported clustering solution for the six populations, suggesting three major ancestral components; however, individuals from each population still exhibited varying degrees of genetic admixture, with no completely pure genetic background observed. This finding corroborates the results from the NJ tree and PCA analyses, collectively revealing the genetic structure characteristics of *E. cyanopodus* populations. The explained variances for PC1 (9.40%) and PC2 (3.40%) were relatively low. In the two-dimensional scatter plot, individuals from the six populations showed a distinctly admixed distribution, with confidence ellipses of the populations overlapping each other. Instead of forming independent clusters corresponding to geographic origins, the populations exhibited a topological structure in which individuals from multiple populations were intermingled. Specifically, close genetic relationships were observed among the HD, ZP, and LS populations, indicating frequent gene flow among populations and highly admixed individual genetic compositions [39]. Fst values between all population pairs ranged from 0.0179 to 0.0422, which were below the significant differentiation threshold of 0.05, indicating that no significant genetic differentiation had occurred among the six populations. The Fst value between the LS and DS populations was relatively higher (0.0422), suggesting that some degree of gene flow restriction may exist between these two populations. Overall, the degree of genetic differentiation among populations was weak, with frequent gene flow or shared germplasm origins, and the level of population genetic structure differentiation had not yet reached a significant level. This also confirms that anthropogenic activities such as artificial introduction and broodstock exchange have broken down geographic barriers and influenced the genetic structure of current cultured populations [40,41]. This finding is consistent with the results of studies using RAPD and Cyt b gene analyses to examine genetic differences among three cultured populations of *E. malabaricus* from Huizhou, Shenzhen, and Zhanjiang, which reported minimal genetic differences and similar genetic backgrounds among cultured populations [42]. Wang et al. [43] also reported no significant genetic differentiation among *E. coioides* populations in Southeast Asia, suggesting that crossbreeding between populations with larger genetic distances could potentially yield heterosis and improve the genetic quality of cultured fish. Artificial reproduction and breeding management have reduced genetic differentiation by promoting gene flow among populations while simultaneously preserving some genetic differences due to independent selection histories at different breeding bases. In studies of cultured populations of turbot (*Scophthalmus maximus*) [44], Atlantic salmon (*Salmo salar*) [45], and red seabream (*Pagrus major*) [46], the main reasons for reduced genetic diversity in cultured populations were founder effects, genetic drift within small populations, and inbreeding accumulation. Due to the limited number of broodstock typically used in aquaculture facilities and the lack of systematic genetic management, allele loss and decreased heterozygosity have occurred. Although the current populations in this study maintained moderate genetic diversity with low inbreeding risk, the weak structural differentiation suggests that moderate gene flow among populations should be maintained. In future stock enhancement or artificial breeding programs, priority should be given to selecting populations with relatively greater genetic distances to maintain and enhance the level of genetic diversity, thereby ensuring the long-term sustainable utilization of germplasm resources.

The selective sweep analysis between the LS and DS populations suggested candidate genes that may be associated with inter-population differences at the genomic level. The selected sweep analysis was performed using a 200 kb sliding window. It is important to note that the estimated half-decay distance of LD in these populations is only 0.4–0.7 kb, meaning that linkage disequilibrium dissipates very quickly across the genome. Consequently, the 200 kb window used for sweep detection is 280–500 times longer than the average distance over which marker pairs remain correlated. This window size was chosen to balance computational efficiency with the coverage of broader genomic regions, but it may reduce resolution for fine-mapping selection footprints. However, the rapid LD decay also implies that strong selection signals are likely to be captured within relatively narrow intervals, and the chosen windows are long enough to detect consistent shifts in Fst and Pi across multiple adjacent LD blocks. Therefore, while the mismatch between window length and LD decay warrants caution, it does not invalidate the overall sweep signals, especially for candidate regions identified by both Fst and Pi methods. A total of 104 selected genes were identified between the two populations, showing substantial differences in chromosomal distribution. The number of selected genes in the DS population (88 genes) was significantly higher than that in the LS population (16 genes), and these genes were mainly concentrated on chromosomes 6, 8, 19, 20, and 21. This could indicate that the DS population may have experienced different selection pressure or management history, but alternative explanations should also be considered. GO enrichment analysis showed that these genes were mainly enriched in functional categories such as actin filament binding, carbonate dehydratase activity, cytoskeletal motor activity, myosin complex, and transcription regulator complex. KEGG enrichment analysis mainly revealed enrichment in pathways including nitrogen metabolism, cardiac muscle contraction, and alanine, aspartate, and glutamate metabolism. Genes related to actin filament binding and cytoskeletal motor activity (Ecy_21G0001040, Ecy_21G0001060, Ecy_21G0001050) are closely associated with muscle development and locomotor capacity, potentially playing important roles in fish adaptation to different water flow conditions and feeding behaviors [47,48]. Carbonate dehydratase activity genes (Ecy_21G0000960, Ecy_21G0000490) are involved in the nitrogen metabolism pathway and are related to ammonia excretion and osmotic pressure regulation, which may reflect selective differences in the metabolic adaptation of fish to different aquaculture environments [49,50]. Enrichment of cardiac muscle contraction-related genes (Ecy_21G0000720) suggests potential adaptive differences in cardiac function and hypoxia tolerance between the two populations [51]. Furthermore, the DS population exhibited a higher number of candidate genes and more diverse functional enrichment. Combined with the relatively higher Fst value between the two populations, this suggests that the DS population may have accumulated more genetic variations—whether through founder effects, drift, or selection—during its breeding history, whereas the LS population may have experienced weaker or different selective pressures. The functional differentiation of these candidate genes indicates that despite the overall low level of genetic differentiation between the two populations, some genomic regions show signals that warrant further investigation. These candidate genes provide hypothesis-generating targets for future studies that incorporate environmental data, pedigree information, or functional validation to disentangle the roles of natural adaptation, artificial selection, and demographic history.

## 5. Conclusions

High-throughput sequencing technology provides abundant molecular markers for fish genetic analysis and offers a powerful tool for studying population genetic patterns. In this study, a large number of SNP loci were identified in *E. cyanopodus*, elucidating the genetic diversity and genetic structure of cultured *E. cyanopodus* populations. Overall, the six populations exhibited moderate genetic diversity with low inbreeding risk. Among them, the LS population showed relatively higher genetic diversity. However, the degree of genetic differentiation among populations was low. Aquaculture management practices such as artificial introduction and broodstock exchange are the main factors leading to unclear population genetic structure. While gene flow reduces population differentiation, it also tends to homogenize genetic backgrounds. The candidate genes identified in this study lay an important foundation for the development of molecular marker-assisted selection for superior strains.

## Figures and Tables

**Table 1:** Sampling locations and information for the six populations of *E. cyanopodus*.

Population	Sampling Location	Number of Samples Collected	Number of Sequenced Samples
ZP	Zhangpu, Fujian (23°55′25″ N, 117°42′20″ E)	32	15
DS	Dongshan, Fujian (23°38′35″ N, 117°24′47″ E)	31	15
SZ	Shenzhen, Guangdong (22°31′23″ N, 114°29′7″ E)	33	15
HD	Huidong, Guangdong (22°43′30″ N, 114°57′7″ E)	30	15
LS	Lingshui, Hainan (18°26′29″ N, 109°59′49″ E)	30	15
DF	Dongfang, Hainan (19°50′60″ N, 109°43′41″ E)	32	15

**Table 2:** Statistics of SNPs and InDels identified in 90 samples. Upstream and downstream indicate that the SNPs are located in both upstream and downstream regions. UTR3 and UTR5 indicate that the SNPs are located in both the 3’ untranslated region and the 5’ untranslated region.

Type		Number
samples		90
SNPs		7,812,666
InDels		1,272,516
intergenic		2,945,649 (33.70%)
intronic		3,980,269
exonic	total	200,057
	synonymous	125,469 (62.72%)
	nonsynonymous	74,588 (37.28%)
splicing		276
upstream		202,114
downstream		181,507
upstream; downstream		17,720
UTR3		221,434
UTR5		61,601
UTR3; UTR5		2039

**Table 3:** Genetic diversity parameters of the six populations.

Population	SNP Density	F	Ho	He	Pi	PIC	Tajima’s D
HD	7.3641/kb	−0.0102 ± 0.1910	0.3388 ± 0.0641	0.3354 ± 0.0000	0.0025 ± 0.0014	0.2604 ± 0.1057	1.1112 ± 1.0636
LS	7.6590/kb	−0.0447 ± 0.2448	0.3684 ± 0.0864	0.3526 ± 0.0001	0.0027 ± 0.0015	0.2742 ± 0.0888	1.3739 ± 0.8242
ZP	7.1635/kb	0.0181 ± 0.1576	0.3511 ± 0.0564	0.3576 ± 0.0001	0.0025 ± 0.0015	0.2775 ± 0.0892	1.4782 ± 0.8788
SZ	7.5731/kb	−0.0092 ± 0.2203	0.3241 ± 0.0708	0.3211 ± 0.0001	0.0024 ± 0.0014	0.2502 ± 0.1106	0.8559 ± 1.0302
DS	6.2093/kb	0.0810 ± 0.0687	0.3415 ± 0.0255	0.3716 ± 0.0001	0.0022 ± 0.0014	0.2859 ± 0.0902	1.6780 ± 0.8563
DF	7.6700/kb	0.0346 ± 0.2353	0.3292 ± 0.0803	0.3410 ± 0.0001	0.0026 ± 0.0014	0.2656 ± 0.0970	1.2000 ± 0.8884

## Data Availability

The original contributions presented in this study are included in the article. Further inquiries can be directed to the corresponding authors.

## Appendix A

**Table A1:** Key statistics of the *E. cyanopodus* reference genome assembly. The reference genome is a gap-free telomere-to-telomere (T2T) chromosome-level genome. The assembly was generated by combining PacBio HiFi, ONT ultralong, Hi-C, and short-read sequencing technologies.

Metric	Value
Total assembly size	1007.08 Mb
Number of chromosomes or scaffolds	4
Contig N50	42.79 Mb
Scaffold N50	42.79 Mb
BUSCO completeness score	99.31%
